# Quantitative Analysis of Seven New Prostate Cancer Biomarkers and the Potential Future of the ‘Biomarker Laboratory’

**DOI:** 10.3390/diagnostics8030049

**Published:** 2018-07-27

**Authors:** Kevin Cao, Callum Arthurs, Ali Atta-ul, Michael Millar, Mariana Beltran, Jochen Neuhaus, Lars-Christian Horn, Rui Henrique, Aamir Ahmed, Christopher Thrasivoulou

**Affiliations:** 1 Prostate Cancer Research Centre at the Centre for Stem Cells and Regenerative Medicine, King’s College London, London WC2R 2LS, UK; kevincaowork@gmail.com (K.C.); callumarthurs@yahoo.co.uk (C.A.); aamir.ahmed@kcl.ac.uk (A.A.); 2 Prostate Cancer Research Centre, University College London, London WC1E 6BT, UK; aliattaul@outlook.com; 3 Queen’s Medical Research Institute, University of Edinburgh, Edinburgh EH8 9YL, UK; mike.millar@ed.ac.uk; 4 Aquila BioMedical, Nine, Edinburgh BioQuarter, 9 Little France Road, Edinburgh EH16 4UX, UK; mariana.beltran@aquila-bm.com; 5 Head of Urology Research Laboratories, University of Leipzig, Department of Urology, Research Laboratory, Liebigstr. 19, Building C, 04103 Leipzig, Germany; jochen.neuhaus@medizin.uni-leipzig.de; 6 Division of Gynecologic, Breast & Perinatal Pathology, University Hospital Leipzig, Liebigstasse 24 D, 04103 Leipzig, Germany; Lars-Christian.Horn@medizin.uni-leipzig.de; 7 Department of Pathology, Portuguese Oncology Institute of Porto, 4200-072 Porto, Portugal; rmhenrique@icbas.up.pt; 8 Department of Pathology and Molecular Immunology, Abel Salazar Institute of Biomedical Sciences, University of Porto, 4099-002 Porto, Portugal; 9 Research Department of Cell and Developmental Biology, The Centre for Cell and Molecular Dynamics, Rockefeller Building, University College London, London WC1E 6BT, UK

**Keywords:** biomarker discovery, tissue microarray, automated workflow, clinical management, morphology-guided analysis

## Abstract

Prostate cancer is the third highest cause of male mortality in the developed world, with the burden of the disease increasing dramatically with demographic change. There are significant limitations to the current diagnostic regimens and no established effective screening modality. To this end, research has discovered hundreds of potential ‘biomarkers’ that may one day be of use in screening, diagnosis or prognostication. However, the barriers to bringing biomarkers to clinical evaluation and eventually into clinical usage have yet to be realised. This is an operational challenge that requires some new thinking and development of paradigms to increase the efficiency of the laboratory process and add ‘value’ to the clinician. Value comes in various forms, whether it be a process that is seamlessly integrated into the hospital laboratory environment or one that can provide additional ‘information’ for the clinical pathologist in terms of risk profiling. We describe, herein, an efficient and tissue-conserving pipeline that uses Tissue Microarrays in a semi-automated process that could, one day, be integrated into the hospital laboratory domain, using seven putative prostate cancer biomarkers for illustration.

## 1. Introduction

### 1.1. Prostate Cancer

Prostate cancer (PCa) is the third highest cause of male mortality in the developed world. In the UK, more than 37,000 cases of PCa are diagnosed per year, making up 25% of all male cancer diagnoses [1,2]. Incidence increases dramatically with age with an estimate of 155 cases per 100,000 men at age 55 and 751 cases per 100,000 men at age 75 [3]. As male lifespan increases, it is predicted that PCa incidence will contribute significantly to the predicted doubling of cancer incidence by 2030 [4].

The one established biochemical test is Prostate-specific antigen (PSA), otherwise known as Kallikrein-related peptidase 3 (KLK3). It is often combined with digital rectal examination and various forms of imaging for both screening and diagnosis. Currently, a PSA cut-off of 4 ng/mL is used to direct patients towards biopsy. This provides a specificity of 93.8% but with a heavily compromised sensitivity of only 20.5% [5,6]. Additionally, PSA can be falsely raised in conditions such as Benign Prostatic Hyperplasia, after infections, or post-ejaculation. Diagnosis is ultimately based on biopsy and histology, which in itself has a 20% false negative rate based on sampling location [7,8,9]. This has prompted debate about alternative biopsy techniques as well as criticism of the histological categorisation of PCa, or Gleason scale, which is based on a pathologist visually scoring the two most dominant areas of malignancy which are summed together. This is particularly problematic for those with the score of 7, where 4 + 3 has worse outcomes than 3 + 4 [10,11,12]. There is also debate about using Magnetic Resonance Imaging (MRI) for screening, but current cost and access are major barriers to widespread implementation.

The primary goal, then, is to find new biomarkers that are more reliable and more predictive than PSA. However, translational research to take putative biomarkers from the laboratory to clinical trial faces major hurdles (elaborated below). A secondary goal, then, for biomarker research must be to find operational strategies that will enhance the speed of biomarker translation, increase the number of donated samples, and in parallel, increase value for clinical pathologists. We envisage this occurring through creating cheaper and more efficient laboratory processes that can be brought nearer to the patients. In this study, we have developed such a pipeline to demonstrate potential gains in biomarker research efficiency using seven putative biomarkers from prostate tissue.

### 1.2. Biomarker Research in Prostate Cancer

An ideal set of disease biomarkers should identify the maximum number of true positive cases (sensitivity) with the fewest false positives (specificity) in a population. Biological molecules (genes, proteins, or biochemicals) rarely show maximum sensitivity with minimal false positive rates for complex diseases such as cancer [13]. The goal of biomarker research is to be able to differentiate between low and high-risk PCa. The National Cancer Institute—Early Detection Research Network (NCI–EDRN) identified five phases of biomarker development from discovery to clinical implementation (Table 1) [14]. Potential biomarkers can be collected from various methods and sources. ‘Invasive’ methods include prostatectomies or biopsy and ‘minimally/non-invasive’ methods include body fluids (serum, plasma, urine, prostatic fluid, and semen). Samples from the former group have the advantage of providing direct analysis of a tumour but are invasive and more challenging in terms of sample preparation. Body fluids have the benefit of being minimally, or non-invasive, and less challenging to prepare but have the drawback of having a lower volume of potential biomarkers hidden amidst a large abundance of proteins that can mask discovery [15]. There are specific benefits and drawbacks to each medium, which is well described in the review by Tonry et al. [16].

Techniques for biomarker discovery include gel-based methods [17] and mass-spectrometry [18]; laboratory evaluation and qualification can involve techniques utilising antibodies, nanotechnology immunosensors, and aptamers [16]. Importantly, when considering biomarker research, one should consider whether the technique of discovery can be adapted to add value to the clinician or pathologist who may, one day, utilise the biomarker in the clinical environment. An example of this approach is the use of non-invasive office-based biomarker tests using urine for the screening of bladder cancer, with one major advantage being rapid results for the General Practitioner and patient [19].

The important fact to clarify in future biomarker research is the benefit of this research to clinicians and to researchers. For clinicians, putative biomarkers derived from body fluids may one day lead to a robust PCa screening programme, whilst biomarkers from tissue provides risk stratification and prediction of recurrence. For the scientist, each type of sample is useful. Body fluids allow for research of systemic associative molecules related to the process of carcinogenesis, whilst histologic material provides an opportunity for morphology-guided analysis focusing on tumour cells, peri-tumour cells, and surrounding tissue architecture.

While the ultimate goal of biomarker research is to find new biomarkers that can be brought to clinical use, the twin problems of a lack of sample donation and inefficiency of testing suggests a new paradigm for biomarker research to tackle these issues is needed. Defining a set of efficient and cost-effective techniques that can be potentially integrated into the hospital-environment may offer clinicians rapid results and solve the problem of a lack of samples for biomarker research. Tissue microarrays (TMA) hold promise in achieving this, and we describe here a pipeline for TMA-based antibody biomarker evaluation that can be used for any of the four phases of biomarker evaluation from discovery to validation.

### 1.3. Tissue Micro Arrays for Discovery

TMA is a powerful and high-throughput technology for the analysis of many paraffin-embedded tissue cores on a single slide. As all cores are treated identically in the preparatory phase of each slide, technical inter-slide variability is reduced, improving standardisation. Many tissue cores can be assembled on a single array leading to a significant economy of scale, reducing the costs of immunohistochemical markers by staining many individual’s samples at the same time. Historically, TMA analysis was conducted using visual measurement of criteria such as stain intensity, distribution and stained-cell count, leading to various comparative scoring systems. Visual examination, however, is the leading limitation in TMA analysis [20]. In the present study, we describe a technique using TMA immunohistochemical staining, captured on a Zeiss Axioscan Z1 (Zeiss, Cambridge, UK) slide scanner, and analysed using a computerised semi-automated image analysis pipeline, producing an objective, replicable and rapid system of biomarker evaluation.

### 1.4. Putative Biomarkers

We tested our pipeline using seven putative biomarkers, which we have studied previously in our laboratory and have existing empirical literature connecting the molecule to cancer and genito-urinary carcinogenesis (Table 2). The potential biomarkers analysed in the present study were: Mitochondrial membrane ATP synthase F1 subunit 1 (ATP5A1), Acyl-coenzyme A Binding protein (ABCP, also known as Diazepam-binding inhibitor (DBI)), 60 kDa Heat Shock Protein (HSP60), Eukaryotic Translation Initiation Factor 3 subunit 1 (IF3EI or EIF3I), Integral Membrane Protein 2B (ITM2B), Myosin Light Chain 6 (MYL6), and Polyadenylate-binding protein 3 (PABPC3).

## 2. Materials and Methods 

### 2.1. Ethics and Specimens

Ethical approval for the use of human prostate tissue in cancer research was granted by the committee of the University College London Joint Research Office and carried out in accordance with the International Committee on Harmonisation of Good Clinical Practice. Archival specimens gathered from open prostatectomies between December 1991 and April 2002 were used. Patients were categorised into two groups. The ‘high-risk malignant’ group (HR) were patients with biochemical relapse within two years (defined as PSA ≥ 0.2 ng mL^−1^), whilst those that required no further cancer therapy and had no biochemical evidence of disease after 3 years were categorised as ‘low-risk malignant’ (LR). Patients who had neoadjuvant chemotherapy or antiandrogen therapy were excluded. Further details of patient selection and inclusion criteria are described in detail elsewhere (https://www.ncbi.nlm.nih.gov/pmc/articles/PMC3752526/). In total 82 patients were included and organised into case pairs containing one HR and one LR, with similar sum Gleason score, preoperative PSA level, and pathological stage subcategory (pT3a or pT3b) at the time of surgery. All samples were examined and classified by a urological pathologist and coded anonymously to avoid bias in tissue array construction, imaging, and analysis.

### 2.2. TMA Design and Construction

The TMA was constructed using formalin-fixed, paraffin-embedded tissue blocks collected from 82 patients who underwent radical prostatectomy, as described previously [64,65,66]. Areas of highest Gleason grade were marked by one pathologist using a haematoxylin and eosin (H + E)-stained 3 μm section of each tissue block. Representative, 0.6 mm diameter and 3 mm depth tissue cores were taken from pre-defined locations using a Beechers MTA 1 (Beechers Instruments, Sun Prairie, WI, USA) manual tissue-arrayer. Four cores were sampled from the two marked tumour locations and four non-malignant cores were sampled from the peripheral zone for equivalence in each specimen. Four replicate TMA blocks were constructed, containing all 82 samples. For positive and negative controls, samples of malignancies in organs other than prostate were included as cores on the tissue block in randomly selected locations. Six to eight micrometre tissue array (TMA) sections were taken for clinical re-characterisation of each core by a urological histopathologist (~78% diagnosed as Gleason grade 3 + 4 or 4 + 3).

### 2.3. Immunohistochemical Staining of Proteins

Staining protocols have been described in detail previously [67]. Standard antigen retrieval was performed on 3 μm sections of each TMA block before individual immunostaining with seven antibodies ATP5A1 (ab14748), DBI (ab16806), HSP60 (ab59457), IF3EI (ab64900), ITM2B (ab89352), MYL6 (ab84349), and PABPC3 (ab68090) all from Abcam (Cambridge, UK) using a 3,3-diaminobenzidine (DAB) staining protocol on an automated Bond MAX™ machine (Leica Biosystems, Milton Keynes, UK) and a Bond polymer detection system kit at high contrast, per manufacturer’s protocols (DS9173). For negative controls, the primary antibody was either replaced with IgG matched controls or omitted.

### 2.4. 3,3-Diaminobenzidine (DAB) Stain Imaging and Signal Quantification

Each TMA slide was imaged on an AxioScan Z1 slide scanner (Zeiss, UK) at 40× magnification and an image of each TMA core was exported for analysis using ImageJ software (NIH, Bethesda, MD, USA). A random selection of tissue cores (*n* = 10 HR, *n* = 10 LR) was used as a training set to select two-pixel value thresholds based on a linear regression model. The first threshold encompassed the quantity of tissue (AmtT) in the image by measuring any non-background pixels; the second threshold encompassed the amount of DAB signal (AmtS) in the image. The quantity of tissue present in each image was calculated using a macro written in ImageJ. Once selected, the same threshold values were applied to every image stained with a single antibody. The amount of DAB signal expressed in each tissue core was calculated as AmtS/AmtT and is used for statistical tests of significance of difference using a Mann-Whitney-U test. Mountain plots were constructed using Origin (OriginLab, Northampton, MA, USA) to illustrate differences in expression between HR and LR cores. Receiver operating characteristics (ROC) curves were constructed in Medcalc software to evaluate the propensity of the protein as a biomarker of malignancy.

### 2.5. Whole Pipeline in Sequence

The entire process described above from initial processing of TMA to eventual results output occurred in a linear process within several days and with further efficiency gains, could be undertaken within a single working day. We assembled four arrays, but this can be changed, and it is quite conceivable that it could be scaled up dramatically, or alternatively, a single core could be processed in each run, reflecting the process of receipt of individual specimens in the hospital pathology laboratory. The statistical techniques are also automated, running in sequence to image capture. The fixed costs of the process are an initial investment in equipment with variable costs being those of antibody stains.

## 3. Results

**Figure 1:**
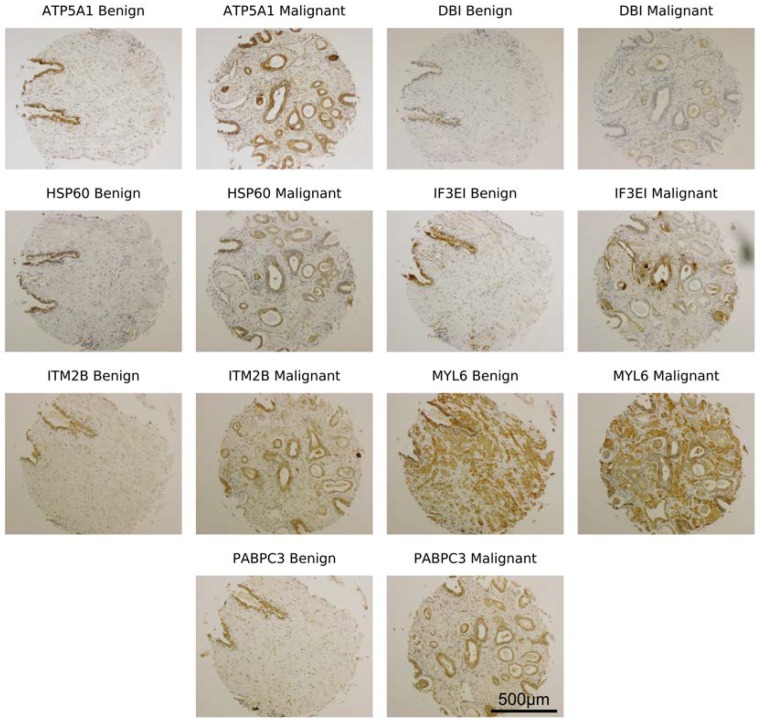
Representative images of a high-risk malignant (HR) and low-risk malignant (LR) prostate cancer tissue core (0.6 mm diameter) taken from sister sections of a tissue array block. Tissue array sections were stained using a 3,3-diaminobenzidine (DAB) protocol for the following proteins (ATP synthase F1 subunit 1 (ATP5A1), Diazepam-binding inhibitor (DBI), 60 kDa Heat Shock Protein (HSP60), IF3EI, Integral Membrane Protein 2B (ITM2B), Myosin Light Chain 6 (MYL6), and Polyadenylate-binding protein 3 (PABPC3)).

Representative images of the seven proteins in TMA core samples are shown in Figure 1. Quantitative analysis of chromophore integrated density performed in ImageJ revealed higher expression of each protein in HR cores compared with LR tissue cores (Table 3).

### Main Findings

**Figure 2:**
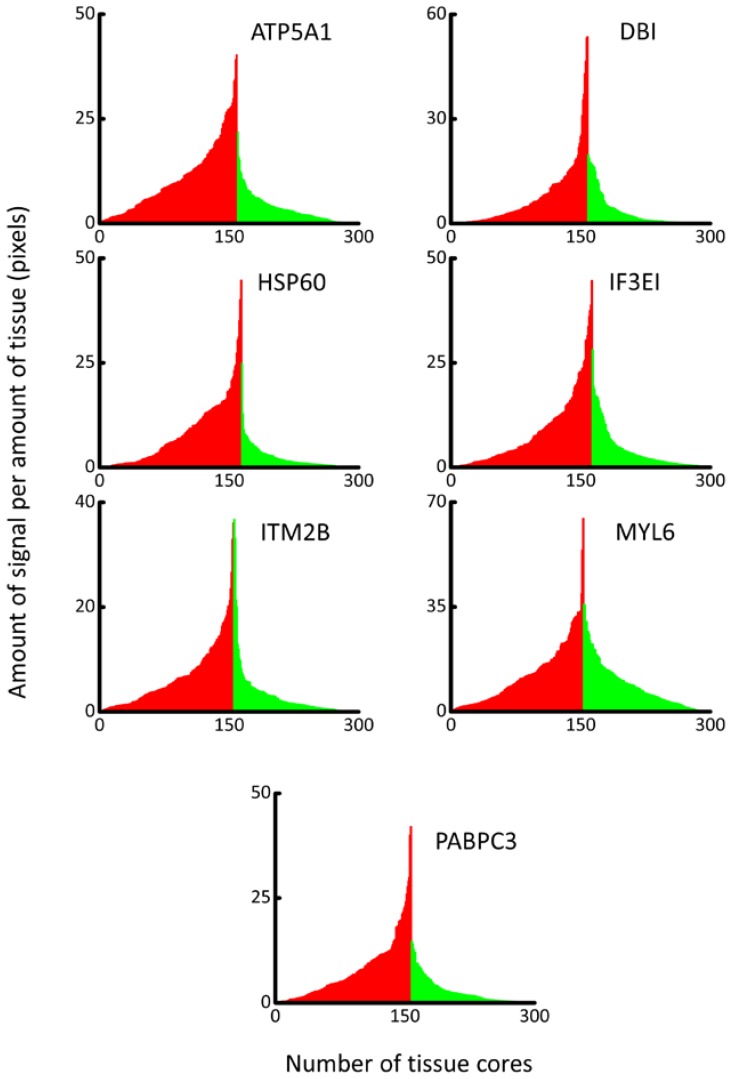
*Mountain Plots*. The total area of DAB stain and the total quantity of tissue present in each tissue core (pixels) was measured using a high throughput, semi-automated protocol using ImageJ software. Mountain plots illustrating the amount of DAB signal per amount of tissue in each tissue core. Red bars represent HR cores arranged in ascending order and green bars are LR tissue cores arranged in descending order. Plots were constructed in Origin (OriginLab, Northampton, MA, USA) software. Significant differences between protein expression in HR and LR tissue cores were observed in all proteins tested: ATP5A1 (*p* < 0.001), DBI (*p* < 0.001), HSP60 (*p* < 0.001), IF3EI (*p* < 0.001), ITM2B (*p* < 0.001), MYL6 (*p* = 0.0012), and PABPC3 (*p* < 0.001).

**Figure 3:**
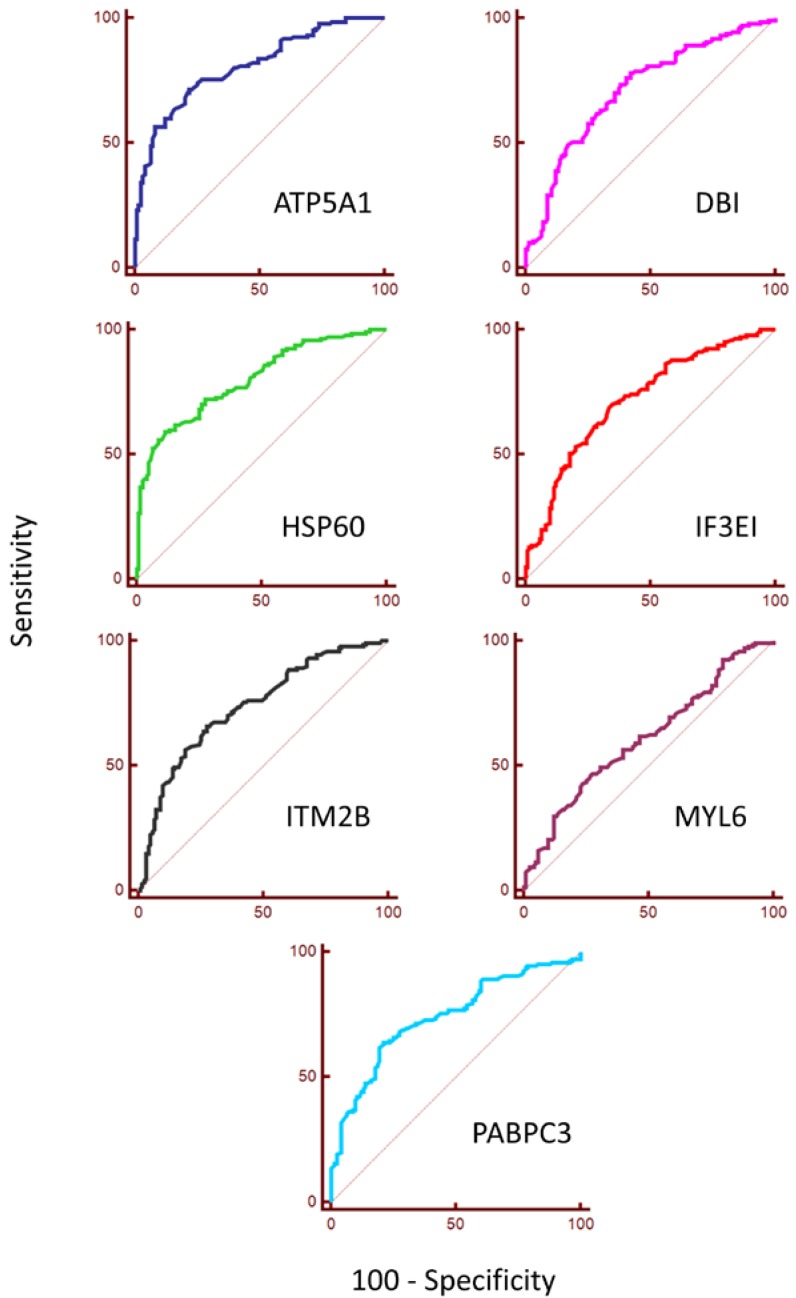
*ROC Curves*. ROC curves for the seven putative biomarkers (ATP5A1, DBI, HSP60, IF3EI, ITM2B, MYL6, and PABPC3) that were tested in this study were constructed to evaluate the diagnostic accuracy of each protein to differentiate between HR and LR tissue cores. Values for AUC, sensitivity and specificity are given in Table 3. Sensitivity and specificity are listed along with respective cut-off values and likelihood ratios in Supplementary Table S1.

Changes in expression have been illustrated in mountain plots (Figure 2), where clear differences in expression can be seen between HR tissue cores (red) and LR tissue cores (green). A Mann–Whitney-*U* test revealed statistically significant differences between HR and LR tissue core expression for ATP5A1 (*p* < 0.001), DBI (*p* < 0.001), HSP60 (*p* < 0.001), IF3EI (*p* < 0.001), ITM2B (*p* < 0.001), MYL6 (*p* = 0.0012), and PABPC3 (*p* < 0.001). Comparing each of the tested proteins for their predictive ability to distinguish between HR and LR tissue cores (Table 3) revealed that ATP5A1 had the highest sensitivity (71.43) and specificity (78.05). ROC curve analysis was carried out to characterise the potential clinical utility of these proteins as biomarkers of malignancy in PCa specimens (Figure 3, Supplementary Table S1). The positive post-test probability ranged from 0.67 to 1.82% (LR+ = 1.463–3.950) and the negative post-test probability ranged between 0.12–0.31% (LR− = 0.2630–0.678). The area under the curve (AUC) of the ROC curve was highest in ATP5A1 (0.806 ± 0.026) and lowest in MYL6 (0.61 ± 0.034) with all other biomarkers demonstrating a range of AUC between these two values. The AUC indicates a greater likelihood of being able to distinguish between disease than other proteins tested, and a higher AUC would generally make a protein a better candidate for putative biomarker. A logistic regression model, performed using MedCalc software, shows an increase in diagnostic performance can be achieved by incorporating data from both ATP5A1 and HSP60. The use of both sets of data resulted in 71.85% of cases being correctly classified (AUC = 0.822 ± 0.025, data not shown in Table 3).

Predictive ability to distinguish between HR and LR tissue cores. Proteins are sorted by descending under the curve (AUC) (area under the receiver operating characteristics (ROC) curve) revealed that ATP5A1 had the highest AUC value (0.806 ± 0.026), followed by HSP60 (0.800 ± 0.026) and PABPC3 (0.740 ± 0.030). Sensitivity and specificity are measured using the Youden index to show the potential effectiveness of the marker. Values calculated from data in Figure 3. NB: Sensitivity and specificity are listed along with respective cut-off values and likelihood ratios in Supplementary Table S1.

## 4. Discussion

### 4.1. Image Analysis and Implications for Future Study

The use of a TMA features several advantages over other techniques in biomarker research, namely each tissue sample has been subjected to identical conditions, enabling simultaneous comparison of potentially several hundred tissue samples [68]. On top of this, a semi-automated, reproducible method through computer analysis of stain intensity increases the objectivity of this system of analysis. Overall, the issues of heterogeneity of staining and subjective visual examination with inter- and intra-observer differences are obviated [69].

### 4.2. Challenges in Prostate Cancer Biomarker Research

At present, there are several bottlenecks to the conversion of putative PCa (indeed any cancer) biomarkers to usable and routinely employed clinical tests. PSA took 10 years to transition from laboratory biomarker to clinical blood test [70], by which point its limitations were well known. Indeed, there have been over 200 PCa biomarkers discovered, but none has completed clinical evaluation [71,72,73,74,75,76,77,78]. There are several barriers to improvements in this process. The largest hurdle is volunteer recruitment in the final stage of clinical evaluation; however, laboratory limitations also exist, including the techniques of testing, the ‘silo-ing’ effect of laboratories, and operational ‘distance’ of the biomarker research facilities. The challenge of increasing clinical volunteer recruitment is beyond the scope of this article, but greater inter-institutional collaboration and ‘visibility’ of biomarker research at primary and secondary care level may enhance the awareness of biomarker research amongst healthy and diseased individuals, potentially increasing recruitment. These actions are likely to require greater scientist, clinician, and policymaker collaboration to realise. While this study is a biomarker evaluation project, the methodology and experience gained from developing our processes allow us to describe operational changes and envisage a paradigm of a more efficient ‘biomarker laboratory’, which we will describe here.

The first technical improvement we would describe is the increased utilisation of tissue-conserving methods such as TMA alongside multiplex-antibody labelling. While this will not increase statistical power, it increases the output of proteomic data from each specimen and conserves tissue.

The second issue is the ‘silo-ing’ effect and resource utilisation of the laboratories themselves. At present, the different steps of biomarker research are often carried out by different specialists often in different locations using instruments, tools, and software packages that are behind walls of commercial licensure. Borrowing theories from industry, the idea of ‘re-engineering’ proposes that specialised work should be centred around outcomes rather than people [79]. The case here being that focusing on the turn-around time of specimens to results and combining specialists into one biomarker-focused team could yield a significantly higher output and more and efficient process. Additionally, new technology such as automated TMA production, staining, microscopy, and iterative machine learning algorithms for image analysis may lead to an operational flow process of less than 24 h from the point of receipt of specimens.

The final aspect of operational improvement is the integration of the biomarker laboratory into the clinical space. This will yield both immediate benefits in terms of reductions in cost and time lost through transport as well as increasing the ‘presence’ of the researchers. This latter point may, through closer clinician-researcher ties, yield greater specimen collection as well as participant recruitment. An eventual possibility is the dual utilisation of techniques such as TMA within the clinical laboratory both for diagnostic histopathological purposes and research simultaneously, yielding gains through greater utilisation and data gathering for biomarker research. This is somewhat analogous to the multi-vehicular assembly line employed in modern automotive manufacturing, where greater yields in outcomes are achieved through reduction of redundancy. All three of these improvements, if realised, would reflect the importance of better planning and integration between researchers and clinicians for the goal of cancer biomarker research [80].

### 4.3. Other Future Scenarios

We have here only described one suggestion of how TMA and imaging techniques used in our laboratory may improve biomarker research efficiency, but other areas of advancement are also of interest. Use of non-invasive body fluid, such as blood and urine, is an exciting mode of increasing volunteer recruitment as is the prospect of bringing the testing to the primary care physician’s office using nanotechnology-based data collection methods [16]. Many paradigms exist for improvements in this field of research but different tissue sources and technologies aside, the main goal must be improvements in the design and execution of the operational process.

## 5. Conclusions

The field of PCa biomarker research is a moving target with new biomarkers discovered constantly but with a long delay in bringing tests to the doctor’s office. Each new discovery prompts research into further biomarkers through their biochemical relationships, with the hope that, eventually, some of these biomarkers can reach the clinical validation stage. The main barriers to achieving this are the speed of laboratory processes and sample donation, which requires volunteer participants. We believe the solution to these limitations is to devise more efficient processes and reduce the barriers to voluntary participation, which will enable the biomarker research process to enter the clinical environment nearer to the patients. To demonstrate a more efficient process, we have identified in the present study, several putative biomarkers using a method that is rapid, objective and specimen conserving. The low-cost technique described has the possibility of being easily scaled up in volume, further refined through automation and importantly, brought closer to the clinicians. All these factors may help contribute towards a more integrated and efficient paradigm of a ‘biomarker laboratory’.

## Figures and Tables

**Table 1:** Phases of biomarker research with burden of tissue and samples (Adapted from Rifai et al. [14]).

Phase	Description	Samples/Tissue	No. of Analytes	No. of Samples
I	Discovery: Identifying candidate biomarkers	Proximal fluids Cells line supernatants Animal model plasma ‘Gold standard’ human plasma (reduced biological variation)	1000s	10s
II	Qualification: Confirm differential abundance of candidates in human plasma	‘Gold standard’ Human plasma (reduced biological variation)	30–100	10s
III	Verification: Begin to assess specificity of candidates	Population-derived human plasma (normal biological variation)	10s	100s
IV/V	Validation and clinical assay development: Establish sensitivity and specificity, assay optimisation	Population-derived human plasma (normal biological variation)	4–10	Many 1000s

**Table 2:** Investigated biomarkers and their empirical evidence relating to cancer pathogenesis.

Putative Biomarkers	
ATP5A1 ATP5A1 is one of two subunits of the mitochondrial ATP synthase, which catalyses ATP synthesis during oxidative phosphorylation. Subunits of ATP synthase are implicated in various malignancies including Clear Cell Renal Cancer, Thyroid, and Prostate malignancy [21]. Research with prostate cancer cells has suggested a role for ATP synthase subunits in protective autophagy as well as binding partners for metastasis-associated membrane proteins [22,23,24].	DBI or ACBP ACBP is a protein that is involved in various functions including steroidogenesis and peptide hormone release [25]. It is overexpressed in lung and hepatocellular carcinoma [26,27] and has been suggested as a predictive marker of outcome after chemotherapy for urothelial cancers [28]. There is also evidence that it has a role as a secreted peptide from prostatic epithelial cells, whose levels rise when exposed to androgens [29,30].
HSP60 Heat shock protein 60 is a mitochondrial protein that assists with the assembly of newly imported proteins in the mitochondria [31]. HSP60 has a role, together with HSP70, in antigen presentation in malignant diseases [32], is enhanced in activity in breast carcinoma [33], and myeloid leukaemia [34]. HSP60 is upregulated in prostate cancer and interacts with cyclophilin D to affect mitochondrial permeability [35,36,37]. It is significantly raised in prostate cancer [38] and has overexpression in associated Gleason score, initial serum PSA levels, and lymph node metastasis as well as androgen independence in locally advanced prostate cancers. Intensity and immunoreactivity of HSP60 predict recurrence and decreased recurrence-free survival [39].	EIF3 complex The Eukaryotic Translation Initiation Factor (EIF) 3 multiprotein complex plays a central role in translation initiation. IF3EI is part of EIF3 complex along with proteins such as EIF3b. Overexpression of EIF3b has been observed in both prostate and bladder tumours [40]. Research suggests other eukaryotic translation initiation factor complexes are involved in tumour advancement in castration-resistant prostate cancer [41,42,43]. Several subunits of the EIF3 protein complex, either through upregulation or downregulation, have been implicated in prostate cancer tumorigenesis [44,45,46,47,48,49]. Gain in function through the addition of the long arm of chromosome 8 is seen commonly along with a gain of EIF function in late-stage prostate cancer in as much as 30% of cases [50,51].
ITM2B ITM2B (formerly BRI2) is a transmembrane protein that processes amyloid, inhibits beta-amyloid peptide aggregation and fibril deposition and induction of neurone outgrowth. It is implicated in neuroblastoma and lymphoma [52,53,54]. In the genitourinary system, it has functional implications in motility and tail formation of spermatozoa [55]. In the realm of cancer research, its gene has been implicated as being lost in mutations of the 13q14 chromosomal arm seen in many sporadic prostate cancers [56].	MYL6 MYL6, one of many myosin alkali light chains that are expressed in smooth muscle and non-muscle tissues. There is evidence for the activation of myosin light chain kinase as a possible mechanism by which PCa may escape dormancy [57] as well as phosphorylation of myosin in the regulation of metastases [58]. Regarding the light chain protein, there is evidence of its importance in maintaining basal lamina matrix integrity; breach of may occur more readily in older individuals, possibly explaining an age-related prostate cancer risk [59].
PABPC family Poly(A)-binding proteins play a role in mRNA stability and translation initiation. Evidence about other proteins in the family, such as PABPC1, suggests it is unregulated in gastric [60], hepatocellular [61], and duodenal [62] carcinomas as well as interacting with the androgen receptor, which is the primary target for prostate cancer treatment [63].	

**Table 3:** Evaluation of seven putative biomarkers.

Protein	Sensitivity	Specificity	Criteria >	AUC
ATP5A1	71.43	78.05	3.37	0.806 ± 0.026
HSP60	59.18	88.62	3.29	0.800 ± 0.026
PABPC3	63.95	78.86	3.26	0.740 ± 0.030
ITM2B	65.99	72.36	3.16	0.738 ± 0.30
IF3EI	68.71	66.61	3.10	0.720 ± 0.031
DBI	78.23	57.72	2.68	0.715 ± 0.031
MYL6	42.26	73.17	3.77	0.610 ± 0.034

## Supplementary Materials

The following are available online at http://www.mdpi.com/2075-4418/8/3/49/s1.
