# Supplementary material for: Quantitative Analysis of Seven New Prostate Cancer Biomarkers and the Potential Future of the ‘Biomarker Laboratory’
Source: Diagnostics (Basel). 2018 Jul 27;8(3):49. doi: 10.3390/diagnostics8030049 (PMC6163663; doi:10.3390/diagnostics8030049)
Supplement: Supplementary file 1 [file diagnostics-08-00049-s001.pdf]

Receiver Operating Characteristics Curve Summary Table

A

ATP5A1

Criterion	Sensitivity	Specificity	+LR	-LR
**≥1.05**	100	0	1	
**>2.19**	100	15.45	1.18	0
**>2.21**	98.64	15.45	1.17	0.088
**>2.35**	98.64	21.14	1.25	0.064
**>2.36**	97.96	21.95	1.26	0.093
**>2.5**	97.96	26.02	1.32	0.078
**>2.56**	96.6	26.02	1.31	0.13
**>2.57**	95.92	26.83	1.31	0.15
**>2.6**	95.92	27.64	1.33	0.15
**>2.61**	95.24	27.64	1.32	0.17
**>2.63**	95.24	28.46	1.33	0.17
**>2.68**	93.2	28.46	1.3	0.24
**>2.69**	93.2	30.89	1.35	0.22
**>2.71**	92.52	30.89	1.34	0.24
**>2.77**	92.52	36.59	1.46	0.2
**>2.79**	91.84	37.4	1.47	0.22
**>2.83**	91.84	40.65	1.55	0.2
**>2.84**	91.16	41.46	1.56	0.21
**>2.89**	87.07	41.46	1.49	0.31
**>2.9**	87.07	42.28	1.51	0.31
**>2.92**	86.39	43.09	1.52	0.32
**>2.93**	85.71	43.9	1.53	0.33
**>2.94**	85.03	43.9	1.52	0.34
**>2.95**	84.35	46.34	1.57	0.34
**>2.96**	83.67	47.15	1.58	0.35
**>2.98**	83.67	50.41	1.69	0.32
**>3.01**	82.31	50.41	1.66	0.35
**>3.03**	82.31	52.85	1.75	0.33
**>3.04**	81.63	54.47	1.79	0.34
**>3.05**	80.95	54.47	1.78	0.35
**>3.1**	80.95	57.72	1.91	0.33
**>3.13**	80.27	59.35	1.97	0.33
**>3.14**	80.27	60.16	2.01	0.33
**>3.15**	79.59	60.98	2.04	0.33
**>3.16**	77.55	62.6	2.07	0.36
**>3.17**	76.87	63.41	2.1	0.36
**>3.18**	76.19	64.23	2.13	0.37
**>3.19**	75.51	65.04	2.16	0.38
**>3.28**	75.51	73.17	2.81	0.33
**>3.29**	74.83	73.98	2.88	0.34
**>3.31**	74.15	74.8	2.94	0.35
**>3.32**	73.47	74.8	2.92	0.35
**>3.33**	72.79	75.61	2.98	0.36
**>3.34**	72.11	76.42	3.06	0.36
**>3.36**	71.43	77.24	3.14	0.37
**>3.37**	71.43	78.05	3.25	0.37
**>3.39**	70.07	78.05	3.19	0.38
**>3.41**	70.07	78.86	3.31	0.38
**>3.42**	68.03	79.67	3.35	0.4
**>3.44**	65.99	79.67	3.25	0.43
**>3.45**	64.63	82.11	3.61	0.43
**>3.46**	63.95	83.74	3.93	0.43
**>3.47**	63.27	84.55	4.1	0.43
**>3.48**	61.22	85.37	4.18	0.45
**>3.5**	59.86	85.37	4.09	0.47
**>3.51**	59.86	87.8	4.91	0.46
**>3.55**	56.46	87.8	4.63	0.5
**>3.59**	56.46	91.87	6.94	0.47
**>3.63**	53.74	91.87	6.61	0.5
**>3.64**	51.7	92.68	7.07	0.52
**>3.67**	48.98	92.68	6.69	0.55
**>3.68**	48.98	93.5	7.53	0.55
**>3.73**	42.18	93.5	6.48	0.62
**>3.74**	41.5	94.31	7.29	0.62
**>3.75**	40.82	95.93	10.04	0.62
**>3.81**	37.41	95.93	9.2	0.65
**>3.82**	36.73	96.75	11.3	0.65
**>3.84**	34.01	96.75	10.46	0.68
**>3.85**	34.01	97.56	13.95	0.68
**>3.96**	25.17	97.56	10.32	0.77
**>3.97**	25.17	98.37	15.48	0.76
**>3.99**	23.13	98.37	14.22	0.78
**>4**	23.13	99.19	28.45	0.78
**>4.2**	11.56	99.19	14.22	0.89
**>4.22**	11.56	100		0.88
**>4.75**	0	100		1

B

HSP60

Criterion	Sensitivity	Specificity	+LR	-LR
≥0.47	100	0	1	
>1.52	100	6.5	1.07	0
>1.56	99.32	6.5	1.06	0.1
>1.6	99.32	8.13	1.08	0.084
>1.63	98.64	8.13	1.07	0.17
>1.91	98.64	13.82	1.14	0.098
>1.92	97.96	13.82	1.14	0.15
>2.03	97.96	16.26	1.17	0.13
>2.04	97.28	17.89	1.18	0.15
>2.2	97.28	23.58	1.27	0.12
>2.24	96.6	24.39	1.28	0.14
>2.25	96.6	26.02	1.31	0.13
>2.3	95.92	26.02	1.3	0.16
>2.42	95.92	33.33	1.44	0.12
>2.43	95.24	33.33	1.43	0.14
>2.44	93.88	34.15	1.43	0.18
>2.48	93.88	36.59	1.48	0.17
>2.49	92.52	36.59	1.46	0.2
>2.53	92.52	39.84	1.54	0.19
>2.54	91.84	39.84	1.53	0.2
>2.56	91.84	41.46	1.57	0.2
>2.57	89.8	41.46	1.53	0.25
>2.58	89.8	42.28	1.56	0.24
>2.59	89.12	44.72	1.61	0.24
>2.61	87.07	44.72	1.58	0.29
>2.62	87.07	45.53	1.6	0.28
>2.63	86.39	46.34	1.61	0.29
>2.64	86.39	47.15	1.63	0.29
>2.65	85.71	47.15	1.62	0.3
>2.67	85.71	48.78	1.67	0.29
>2.68	83.67	49.59	1.66	0.33
>2.69	82.31	52.85	1.75	0.33
>2.7	81.63	52.85	1.73	0.35
>2.71	80.95	54.47	1.78	0.35
>2.72	78.23	55.28	1.75	0.39
>2.73	76.87	56.1	1.75	0.41
>2.78	76.87	60.16	1.93	0.38
>2.79	76.19	60.98	1.95	0.39
>2.8	75.51	61.79	1.98	0.4
>2.81	75.51	63.41	2.06	0.39
>2.82	74.83	63.41	2.05	0.4
>2.84	74.15	64.23	2.07	0.4
>2.85	74.15	65.04	2.12	0.4
>2.86	72.79	65.85	2.13	0.41
>2.89	72.79	68.29	2.3	0.4
>2.91	72.11	68.29	2.27	0.41
>2.94	72.11	72.36	2.61	0.39
>2.96	70.07	72.36	2.53	0.41
>2.97	70.07	73.98	2.69	0.4
>3.01	68.03	73.98	2.61	0.43
>3.02	68.03	74.8	2.7	0.43
>3.07	64.63	74.8	2.56	0.47
>3.08	64.63	76.42	2.74	0.46
>3.09	63.95	77.24	2.81	0.47
>3.1	63.27	78.86	2.99	0.47
>3.12	63.27	80.49	3.24	0.46
>3.15	62.59	82.11	3.5	0.46
>3.16	61.9	82.93	3.63	0.46
>3.17	61.9	84.55	4.01	0.45
>3.2	59.86	84.55	3.88	0.47
>3.25	59.86	86.99	4.6	0.46
>3.26	59.18	86.99	4.55	0.47
>3.29	59.18	88.62	5.2	0.46
>3.3	58.5	88.62	5.14	0.47
>3.33	56.46	89.43	5.34	0.49
>3.36	55.78	90.24	5.72	0.49
>3.37	55.78	91.06	6.24	0.49
>3.4	54.42	91.06	6.09	0.5
>3.41	53.74	91.87	6.61	0.5
>3.42	52.38	92.68	7.16	0.51
>3.43	52.38	93.5	8.05	0.51
>3.44	51.02	93.5	7.84	0.52
>3.45	48.98	94.31	8.61	0.54
>3.46	48.3	94.31	8.49	0.55
>3.47	46.94	95.12	9.62	0.56
>3.54	42.18	95.12	8.65	0.61
>3.56	41.5	95.93	10.21	0.61
>3.58	40.14	95.93	9.87	0.62
>3.59	40.14	96.75	12.34	0.62
>3.6	39.46	97.56	16.18	0.62
>3.65	36.73	97.56	15.06	0.65
>3.66	36.73	98.37	22.59	0.64
>3.84	26.53	98.37	16.32	0.75
>3.86	26.53	99.19	32.63	0.74
>4.31	4.08	99.19	5.02	0.97
>4.32	4.08	100		0.96
>4.87	0	100		1

C

PABPC3

Criterion	Sensitivity	Specificity	+LR	-LR
**≥0**	100	0	1	
**>0.62**	97.28	0	0.97	
**>1.68**	97.28	3.25	1.01	0.84
**>1.78**	96.6	3.25	1	1.05
**>1.84**	96.6	4.07	1.01	0.84
**>1.85**	95.92	4.07	1	1
**>2.06**	95.92	11.38	1.08	0.36
**>2.07**	95.24	12.2	1.08	0.39
**>2.18**	95.24	17.07	1.15	0.28
**>2.21**	94.56	17.89	1.15	0.3
**>2.3**	94.56	21.14	1.2	0.26
**>2.31**	93.88	21.14	1.19	0.29
**>2.33**	93.88	21.95	1.2	0.28
**>2.34**	93.2	21.95	1.19	0.31
**>2.36**	92.52	22.76	1.2	0.33
**>2.37**	91.84	22.76	1.19	0.36
**>2.38**	91.16	23.58	1.19	0.38
**>2.39**	90.48	25.2	1.21	0.38
**>2.48**	90.48	30.89	1.31	0.31
**>2.5**	89.8	32.52	1.33	0.31
**>2.54**	89.12	33.33	1.34	0.33
**>2.61**	89.12	39.02	1.46	0.28
**>2.62**	88.44	39.84	1.47	0.29
**>2.67**	84.35	39.84	1.4	0.39
**>2.7**	84.35	40.65	1.42	0.38
**>2.71**	83.67	40.65	1.41	0.4
**>2.72**	82.99	41.46	1.42	0.41
**>2.73**	81.63	42.28	1.41	0.43
**>2.74**	80.95	42.28	1.4	0.45
**>2.76**	80.95	43.09	1.42	0.44
**>2.79**	79.59	43.09	1.4	0.47
**>2.8**	78.91	45.53	1.45	0.46
**>2.82**	77.55	45.53	1.42	0.49
**>2.83**	77.55	46.34	1.45	0.48
**>2.86**	76.87	46.34	1.43	0.5
**>2.9**	76.87	52.85	1.63	0.44
**>2.91**	76.19	52.85	1.62	0.45
**>2.92**	75.51	53.66	1.63	0.46
**>2.95**	75.51	56.1	1.72	0.44
**>2.96**	74.83	56.91	1.74	0.44
**>2.98**	74.15	57.72	1.75	0.45
**>2.99**	73.47	57.72	1.74	0.46
**>3**	72.79	59.35	1.79	0.46
**>3.03**	72.79	62.6	1.95	0.43
**>3.04**	72.11	63.41	1.97	0.44
**>3.05**	71.43	65.04	2.04	0.44
**>3.06**	71.43	65.85	2.09	0.43
**>3.07**	70.75	65.85	2.07	0.44
**>3.08**	70.75	66.67	2.12	0.44
**>3.09**	70.07	68.29	2.21	0.44
**>3.1**	69.39	69.11	2.25	0.44
**>3.11**	69.39	69.92	2.31	0.44
**>3.12**	68.71	70.73	2.35	0.44
**>3.13**	68.71	71.54	2.41	0.44
**>3.14**	68.03	72.36	2.46	0.44
**>3.16**	66.67	72.36	2.41	0.46
**>3.17**	66.67	73.17	2.48	0.46
**>3.18**	65.99	73.98	2.54	0.46
**>3.19**	65.99	74.8	2.62	0.45
**>3.2**	65.31	74.8	2.59	0.46
**>3.21**	64.63	75.61	2.65	0.47
**>3.22**	64.63	76.42	2.74	0.46
**>3.23**	63.95	76.42	2.71	0.47
**>3.26**	63.95	78.86	3.03	0.46
**>3.27**	63.27	78.86	2.99	0.47
**>3.28**	61.9	80.49	3.17	0.47
**>3.33**	56.46	80.49	2.89	0.54
**>3.34**	55.1	81.3	2.95	0.55
**>3.35**	54.42	81.3	2.91	0.56
**>3.36**	53.74	82.11	3	0.56
**>3.39**	49.66	82.11	2.78	0.61
**>3.4**	48.98	83.74	3.01	0.61
**>3.42**	48.3	83.74	2.97	0.62
**>3.43**	48.3	84.55	3.13	0.61
**>3.44**	47.62	85.37	3.25	0.61
**>3.45**	47.62	86.18	3.45	0.61
**>3.46**	46.26	86.18	3.35	0.62
**>3.47**	45.58	86.99	3.5	0.63
**>3.49**	44.22	86.99	3.4	0.64
**>3.5**	44.22	87.8	3.63	0.64
**>3.51**	42.18	88.62	3.71	0.65
**>3.52**	42.18	89.43	3.99	0.65
**>3.54**	41.5	89.43	3.93	0.65
**>3.55**	40.82	90.24	4.18	0.66
**>3.59**	36.73	90.24	3.77	0.7
**>3.6**	36.73	91.06	4.11	0.69
**>3.62**	36.05	91.06	4.03	0.7
**>3.64**	36.05	92.68	4.93	0.69
**>3.65**	35.37	93.5	5.44	0.69
**>3.66**	34.69	93.5	5.33	0.7
**>3.68**	34.01	94.31	5.98	0.7
**>3.69**	31.97	95.93	7.87	0.71
**>3.82**	19.73	95.93	4.85	0.84
**>3.83**	19.05	97.56	7.81	0.83
**>3.86**	15.65	97.56	6.41	0.86
**>3.87**	15.65	98.37	9.62	0.86
**>3.89**	14.97	98.37	9.2	0.86
**>3.92**	14.29	99.19	17.57	0.86
**>3.95**	13.61	100		0.86
**>4.8**	0	100		1

D

ITM2B

Criterion	Sensitivity	Specificity	+LR	-LR
**≥1.31**	100	0	1	
**>1.67**	100	2.44	1.03	0
**>1.74**	99.32	3.25	1.03	0.21
**>2.07**	99.32	8.94	1.09	0.076
**>2.08**	98.64	8.94	1.08	0.15
**>2.11**	98.64	9.76	1.09	0.14
**>2.12**	97.96	9.76	1.09	0.21
**>2.26**	97.96	18.7	1.2	0.11
**>2.27**	97.28	18.7	1.2	0.15
**>2.36**	97.28	19.51	1.21	0.14
**>2.38**	95.92	19.51	1.19	0.21
**>2.45**	95.92	24.39	1.27	0.17
**>2.48**	95.24	26.02	1.29	0.18
**>2.5**	94.56	26.83	1.29	0.2
**>2.53**	94.56	28.46	1.32	0.19
**>2.54**	93.2	28.46	1.3	0.24
**>2.59**	93.2	31.71	1.36	0.21
**>2.61**	92.52	32.52	1.37	0.23
**>2.64**	89.8	32.52	1.33	0.31
**>2.66**	89.12	34.15	1.35	0.32
**>2.68**	89.12	36.59	1.41	0.3
**>2.69**	88.44	36.59	1.39	0.32
**>2.71**	88.44	39.84	1.47	0.29
**>2.72**	87.07	40.65	1.47	0.32
**>2.76**	84.35	40.65	1.42	0.38
**>2.77**	83.67	41.46	1.43	0.39
**>2.78**	82.31	43.9	1.47	0.4
**>2.79**	81.63	44.72	1.48	0.41
**>2.8**	80.27	46.34	1.5	0.43
**>2.81**	78.91	47.97	1.52	0.44
**>2.82**	78.23	48.78	1.53	0.45
**>2.83**	77.55	48.78	1.51	0.46
**>2.84**	76.19	50.41	1.54	0.47
**>2.89**	76.19	56.1	1.74	0.42
**>2.91**	75.51	56.91	1.75	0.43
**>2.92**	75.51	57.72	1.79	0.42
**>2.93**	73.47	59.35	1.81	0.45
**>2.94**	73.47	60.16	1.84	0.44
**>2.95**	72.79	60.98	1.87	0.45
**>2.96**	72.11	61.79	1.89	0.45
**>2.97**	71.43	61.79	1.87	0.46
**>2.98**	71.43	62.6	1.91	0.46
**>2.99**	70.75	62.6	1.89	0.47
**>3.05**	70.07	64.23	1.96	0.47
**>3.08**	68.71	64.23	1.92	0.49
**>3.1**	67.35	65.04	1.93	0.5
**>3.13**	67.35	69.92	2.24	0.47
**>3.14**	66.67	70.73	2.28	0.47
**>3.15**	65.99	71.54	2.32	0.48
**>3.16**	65.99	72.36	2.39	0.47
**>3.17**	63.95	72.36	2.31	0.5
**>3.19**	63.95	73.98	2.46	0.49
**>3.2**	63.27	73.98	2.43	0.5
**>3.21**	61.9	74.8	2.46	0.51
**>3.22**	60.54	74.8	2.4	0.53
**>3.23**	58.5	75.61	2.4	0.55
**>3.24**	57.82	78.86	2.74	0.53
**>3.25**	57.14	79.67	2.81	0.54
**>3.26**	56.46	81.3	3.02	0.54
**>3.28**	53.74	81.3	2.87	0.57
**>3.29**	53.06	82.93	3.11	0.57
**>3.3**	51.7	82.93	3.03	0.58
**>3.31**	49.66	83.74	3.05	0.6
**>3.32**	49.66	85.37	3.39	0.59
**>3.33**	48.98	85.37	3.35	0.6
**>3.34**	48.98	86.18	3.54	0.59
**>3.39**	45.58	86.18	3.3	0.63
**>3.41**	44.9	86.99	3.45	0.63
**>3.42**	43.54	87.8	3.57	0.64
**>3.43**	43.54	88.62	3.83	0.64
**>3.45**	42.18	90.24	4.32	0.64
**>3.49**	38.1	90.24	3.9	0.69
**>3.5**	37.41	91.06	4.18	0.69
**>3.52**	32.65	91.06	3.65	0.74
**>3.56**	32.65	92.68	4.46	0.73
**>3.6**	29.25	92.68	4	0.76
**>3.62**	28.57	93.5	4.39	0.76
**>3.68**	24.49	93.5	3.77	0.81
**>3.7**	23.81	94.31	4.18	0.81
**>3.72**	23.13	94.31	4.06	0.82
**>3.73**	22.45	95.12	4.6	0.82
**>3.78**	18.37	95.12	3.77	0.86
**>3.82**	17.69	95.93	4.35	0.86
**>3.86**	14.97	95.93	3.68	0.89
**>3.87**	14.97	96.75	4.6	0.88
**>4.13**	4.76	96.75	1.46	0.98
**>4.16**	4.08	97.56	1.67	0.98
**>4.17**	3.4	97.56	1.39	0.99
**>4.2**	2.72	98.37	1.67	0.99
**>4.38**	1.36	98.37	0.84	1
**>4.56**	0.68	99.19	0.84	1
**>4.64**	0	99.19	0	1.01
**>4.66**	0	100		1

E

IF3EI

Criterion	Sensitivity	Specificity	+LR	-LR
**≥1.33**	100	0	1	
**>1.84**	100	5.69	1.06	0
**>1.86**	99.32	5.69	1.05	0.12
**>1.9**	99.32	6.5	1.06	0.1
**>1.91**	98.64	7.32	1.06	0.19
**>1.93**	97.96	7.32	1.06	0.28
**>2.07**	97.96	11.38	1.11	0.18
**>2.08**	97.28	11.38	1.1	0.24
**>2.17**	97.28	13.82	1.13	0.2
**>2.19**	96.6	13.82	1.12	0.25
**>2.32**	96.6	16.26	1.15	0.21
**>2.33**	95.92	17.07	1.16	0.24
**>2.35**	95.92	17.89	1.17	0.23
**>2.36**	95.24	19.51	1.18	0.24
**>2.37**	95.24	20.33	1.2	0.23
**>2.38**	93.88	20.33	1.18	0.3
**>2.41**	93.88	22.76	1.22	0.27
**>2.43**	92.52	22.76	1.2	0.33
**>2.48**	92.52	27.64	1.28	0.27
**>2.49**	91.84	27.64	1.27	0.3
**>2.5**	91.84	28.46	1.28	0.29
**>2.52**	91.16	29.27	1.29	0.3
**>2.56**	91.16	30.89	1.32	0.29
**>2.57**	90.48	31.71	1.32	0.3
**>2.58**	89.8	31.71	1.31	0.32
**>2.59**	89.8	32.52	1.33	0.31
**>2.6**	89.12	33.33	1.34	0.33
**>2.62**	88.44	33.33	1.33	0.35
**>2.63**	88.44	34.15	1.34	0.34
**>2.64**	87.76	35.77	1.37	0.34
**>2.76**	87.76	41.46	1.5	0.3
**>2.77**	87.07	42.28	1.51	0.31
**>2.78**	86.39	43.9	1.54	0.31
**>2.8**	82.99	43.9	1.48	0.39
**>2.82**	82.99	45.53	1.52	0.37
**>2.84**	81.63	47.97	1.57	0.38
**>2.85**	80.95	47.97	1.56	0.4
**>2.86**	78.91	48.78	1.54	0.43
**>2.87**	78.91	50.41	1.59	0.42
**>2.88**	78.23	51.22	1.6	0.43
**>2.9**	76.19	51.22	1.56	0.46
**>2.93**	76.19	53.66	1.64	0.44
**>2.94**	75.51	54.47	1.66	0.45
**>2.95**	74.15	55.28	1.66	0.47
**>2.97**	74.15	57.72	1.75	0.45
**>2.98**	73.47	58.54	1.77	0.45
**>3.01**	73.47	60.16	1.84	0.44
**>3.02**	72.11	60.98	1.85	0.46
**>3.03**	71.43	62.6	1.91	0.46
**>3.04**	70.75	62.6	1.89	0.47
**>3.07**	70.75	63.41	1.93	0.46
**>3.08**	70.07	65.04	2	0.46
**>3.09**	69.39	65.85	2.03	0.46
**>3.1**	68.71	66.67	2.06	0.47
**>3.13**	65.99	67.48	2.03	0.5
**>3.14**	65.31	67.48	2.01	0.51
**>3.15**	63.95	68.29	2.02	0.53
**>3.17**	62.59	69.11	2.03	0.54
**>3.18**	62.59	70.73	2.14	0.53
**>3.19**	61.9	71.54	2.18	0.53
**>3.2**	61.22	71.54	2.15	0.54
**>3.21**	61.22	72.36	2.21	0.54
**>3.23**	59.86	73.17	2.23	0.55
**>3.24**	57.14	74.8	2.27	0.57
**>3.25**	56.46	75.61	2.31	0.58
**>3.27**	54.42	75.61	2.23	0.6
**>3.31**	54.42	77.24	2.39	0.59
**>3.33**	53.74	78.05	2.45	0.59
**>3.34**	53.06	79.67	2.61	0.59
**>3.36**	51.7	79.67	2.54	0.61
**>3.37**	50.34	81.3	2.69	0.61
**>3.39**	50.34	82.11	2.81	0.6
**>3.42**	45.58	82.11	2.55	0.66
**>3.44**	45.58	83.74	2.8	0.65
**>3.45**	44.9	83.74	2.76	0.66
**>3.48**	44.9	84.55	2.91	0.65
**>3.49**	44.22	84.55	2.86	0.66
**>3.5**	44.22	85.37	3.02	0.65
**>3.56**	42.86	85.37	2.93	0.67
**>3.57**	41.5	86.18	3	0.68
**>3.58**	40.82	86.18	2.95	0.69
**>3.61**	40.14	86.99	3.09	0.69
**>3.63**	38.78	87.8	3.18	0.7
**>3.64**	37.41	87.8	3.07	0.71
**>3.65**	36.73	88.62	3.23	0.71
**>3.72**	32.65	88.62	2.87	0.76
**>3.74**	31.29	89.43	2.96	0.77
**>3.77**	28.57	89.43	2.7	0.8
**>3.79**	28.57	90.24	2.93	0.79
**>3.85**	22.45	90.24	2.3	0.86
**>3.87**	21.77	91.87	2.68	0.85
**>3.89**	19.73	91.87	2.43	0.87
**>3.93**	19.73	93.5	3.03	0.86
**>3.99**	16.33	93.5	2.51	0.89
**>4**	16.33	94.31	2.87	0.89
**>4.01**	14.97	94.31	2.63	0.9
**>4.03**	14.29	95.12	2.93	0.9
**>4.04**	13.61	95.93	3.35	0.9
**>4.05**	13.61	97.56	5.58	0.89
**>4.08**	12.93	97.56	5.3	0.89
**>4.11**	12.93	98.37	7.95	0.89
**>4.13**	12.24	98.37	7.53	0.89
**>4.14**	11.56	99.19	14.22	0.89
**>4.35**	4.76	99.19	5.86	0.96
**>4.42**	4.76	100		0.95
**>4.87**	0	100		1

F

DBI

Criterion	Sensitivity	Specificity	+LR	-LR
**>1.02**	99.32	2.44	1.02	0.28
**>1.12**	98.64	2.44	1.01	0.56
**>1.4**	98.64	5.69	1.05	0.24
**>1.44**	97.96	5.69	1.04	0.36
**>1.71**	97.96	10.57	1.1	0.19
**>1.74**	97.28	10.57	1.09	0.26
**>1.8**	97.28	13.01	1.12	0.21
**>1.83**	96.6	13.01	1.11	0.26
**>1.86**	96.6	13.82	1.12	0.25
**>1.92**	95.92	13.82	1.11	0.3
**>1.94**	95.92	14.63	1.12	0.28
**>1.95**	94.56	14.63	1.11	0.37
**>2.04**	94.56	17.07	1.14	0.32
**>2.05**	93.88	17.89	1.14	0.34
**>2.07**	93.88	19.51	1.17	0.31
**>2.08**	93.2	19.51	1.16	0.35
**>2.11**	93.2	21.95	1.19	0.31
**>2.13**	91.84	21.95	1.18	0.37
**>2.21**	91.84	25.2	1.23	0.32
**>2.22**	91.16	25.2	1.22	0.35
**>2.24**	90.48	26.02	1.22	0.37
**>2.27**	90.48	27.64	1.25	0.34
**>2.3**	89.8	27.64	1.24	0.37
**>2.31**	89.8	28.46	1.26	0.36
**>2.34**	89.12	28.46	1.25	0.38
**>2.41**	89.12	35.77	1.39	0.3
**>2.43**	87.76	35.77	1.37	0.34
**>2.44**	86.39	37.4	1.38	0.36
**>2.45**	86.39	39.02	1.42	0.35
**>2.46**	85.71	39.02	1.41	0.37
**>2.48**	85.71	39.84	1.42	0.36
**>2.52**	82.99	39.84	1.38	0.43
**>2.53**	82.31	40.65	1.39	0.44
**>2.55**	82.31	45.53	1.51	0.39
**>2.56**	81.63	45.53	1.5	0.4
**>2.57**	80.95	46.34	1.51	0.41
**>2.62**	80.95	51.22	1.66	0.37
**>2.63**	80.27	51.22	1.65	0.39
**>2.64**	78.91	53.66	1.7	0.39
**>2.66**	78.91	56.1	1.8	0.38
**>2.67**	78.23	56.91	1.82	0.38
**>2.68**	78.23	57.72	1.85	0.38
**>2.69**	76.19	57.72	1.8	0.41
**>2.72**	76.19	59.35	1.87	0.4
**>2.77**	74.15	59.35	1.82	0.44
**>2.78**	73.47	60.16	1.84	0.44
**>2.79**	73.47	61.79	1.92	0.43
**>2.81**	72.79	61.79	1.9	0.44
**>2.82**	72.11	62.6	1.93	0.45
**>2.87**	70.07	62.6	1.87	0.48
**>2.9**	70.07	64.23	1.96	0.47
**>2.94**	66.67	64.23	1.86	0.52
**>2.97**	66.67	65.85	1.95	0.51
**>2.98**	65.99	67.48	2.03	0.5
**>3.02**	63.95	67.48	1.97	0.53
**>3.04**	63.27	69.11	2.05	0.53
**>3.05**	63.27	69.92	2.1	0.53
**>3.07**	62.59	69.92	2.08	0.54
**>3.08**	61.9	70.73	2.12	0.54
**>3.1**	61.9	71.54	2.18	0.53
**>3.11**	59.86	72.36	2.17	0.55
**>3.12**	59.86	73.17	2.23	0.55
**>3.15**	57.82	73.17	2.16	0.58
**>3.16**	57.82	74.8	2.29	0.56
**>3.2**	55.1	74.8	2.19	0.6
**>3.21**	54.42	75.61	2.23	0.6
**>3.22**	53.74	75.61	2.2	0.61
**>3.23**	51.7	77.24	2.27	0.63
**>3.26**	50.34	77.24	2.21	0.64
**>3.3**	50.34	81.3	2.69	0.61
**>3.31**	49.66	82.93	2.91	0.61
**>3.33**	47.62	83.74	2.93	0.63
**>3.35**	45.58	83.74	2.8	0.65
**>3.36**	45.58	84.55	2.95	0.64
**>3.37**	44.9	84.55	2.91	0.65
**>3.39**	44.9	85.37	3.07	0.65
**>3.41**	44.22	85.37	3.02	0.65
**>3.42**	43.54	86.18	3.15	0.66
**>3.45**	42.18	86.18	3.05	0.67
**>3.47**	40.14	86.99	3.09	0.69
**>3.48**	38.78	86.99	2.98	0.7
**>3.49**	38.78	87.8	3.18	0.7
**>3.6**	34.01	87.8	2.79	0.75
**>3.61**	33.33	88.62	2.93	0.75
**>3.62**	31.97	89.43	3.03	0.76
**>3.64**	29.25	89.43	2.77	0.79
**>3.65**	29.25	91.06	3.27	0.78
**>3.84**	19.73	91.06	2.21	0.88
**>3.85**	18.37	91.87	2.26	0.89
**>3.86**	18.37	92.68	2.51	0.88
**>3.95**	15.65	92.68	2.14	0.91
**>3.96**	14.97	93.5	2.3	0.91
**>4**	12.93	93.5	1.99	0.93
**>4.01**	12.24	94.31	2.15	0.93
**>4.02**	11.56	95.12	2.37	0.93
**>4.03**	10.88	95.93	2.68	0.93
**>4.04**	10.88	96.75	3.35	0.92
**>4.05**	10.2	96.75	3.14	0.93
**>4.06**	10.2	98.37	6.28	0.91
**>4.09**	9.52	98.37	5.86	0.92
**>4.11**	8.16	99.19	10.04	0.93
**>4.14**	7.48	99.19	9.2	0.93
**>4.15**	7.48	100		0.93
**>5.09**	0	100		1

G

MYL6

Criterion	Sensitivity	Specificity	+LR	-LR
**≥1.37**	100	0	1	
**>1.37**	99.32	0	0.99	
**>2.12**	99.32	7.32	1.07	0.093
**>2.26**	98.64	7.32	1.06	0.19
**>2.32**	98.64	8.13	1.07	0.17
**>2.33**	97.96	8.13	1.07	0.25
**>2.46**	97.96	9.76	1.09	0.21
**>2.49**	97.28	9.76	1.08	0.28
**>2.52**	97.28	11.38	1.1	0.24
**>2.63**	95.92	11.38	1.08	0.36
**>2.65**	95.92	13.82	1.11	0.3
**>2.68**	95.24	14.63	1.12	0.33
**>2.69**	95.24	15.45	1.13	0.31
**>2.73**	94.56	16.26	1.13	0.33
**>2.77**	93.2	16.26	1.11	0.42
**>2.8**	93.2	17.07	1.12	0.4
**>2.81**	92.52	17.89	1.13	0.42
**>2.84**	92.52	20.33	1.16	0.37
**>2.91**	89.12	20.33	1.12	0.54
**>2.92**	88.44	21.95	1.13	0.53
**>2.95**	86.39	21.95	1.11	0.62
**>2.99**	85.03	22.76	1.1	0.66
**>3.03**	82.31	22.76	1.07	0.78
**>3.04**	82.31	24.39	1.09	0.73
**>3.05**	80.95	24.39	1.07	0.78
**>3.06**	80.27	25.2	1.07	0.78
**>3.07**	79.59	25.2	1.06	0.81
**>3.09**	79.59	27.64	1.1	0.74
**>3.1**	78.91	28.46	1.1	0.74
**>3.11**	78.23	29.27	1.11	0.74
**>3.12**	78.23	30.08	1.12	0.72
**>3.13**	77.55	30.08	1.11	0.75
**>3.14**	77.55	31.71	1.14	0.71
**>3.15**	76.87	32.52	1.14	0.71
**>3.18**	74.83	32.52	1.11	0.77
**>3.19**	74.83	33.33	1.12	0.76
**>3.2**	74.15	34.15	1.13	0.76
**>3.21**	74.15	34.96	1.14	0.74
**>3.22**	72.79	34.96	1.12	0.78
**>3.23**	72.11	36.59	1.14	0.76
**>3.25**	71.43	38.21	1.16	0.75
**>3.27**	70.75	39.02	1.16	0.75
**>3.28**	69.39	39.84	1.15	0.77
**>3.3**	69.39	41.46	1.19	0.74
**>3.33**	67.35	41.46	1.15	0.79
**>3.34**	66.67	42.28	1.15	0.79
**>3.35**	65.99	43.09	1.16	0.79
**>3.36**	65.31	43.9	1.16	0.79
**>3.37**	65.31	44.72	1.18	0.78
**>3.38**	64.63	45.53	1.19	0.78
**>3.39**	63.95	46.34	1.19	0.78
**>3.4**	62.59	47.15	1.18	0.79
**>3.42**	62.59	49.59	1.24	0.75
**>3.43**	61.9	51.22	1.27	0.74
**>3.46**	61.9	53.66	1.34	0.71
**>3.5**	59.18	53.66	1.28	0.76
**>3.53**	59.18	55.28	1.32	0.74
**>3.55**	58.5	55.28	1.31	0.75
**>3.56**	57.82	56.1	1.32	0.75
**>3.57**	57.14	56.1	1.3	0.76
**>3.58**	56.46	56.91	1.31	0.77
**>3.6**	56.46	60.16	1.42	0.72
**>3.63**	53.06	60.16	1.33	0.78
**>3.64**	53.06	60.98	1.36	0.77
**>3.65**	52.38	62.6	1.4	0.76
**>3.66**	51.7	64.23	1.45	0.75
**>3.67**	51.02	65.04	1.46	0.75
**>3.68**	49.66	66.67	1.49	0.76
**>3.7**	49.66	69.11	1.61	0.73
**>3.73**	48.3	69.11	1.56	0.75
**>3.74**	46.94	70.73	1.6	0.75
**>3.76**	46.94	72.36	1.7	0.73
**>3.77**	46.26	73.17	1.72	0.73
**>3.78**	44.9	73.98	1.73	0.74
**>3.79**	44.22	74.8	1.75	0.75
**>3.8**	43.54	74.8	1.73	0.75
**>3.81**	42.86	76.42	1.82	0.75
**>3.83**	41.5	77.24	1.82	0.76
**>3.84**	39.46	77.24	1.73	0.78
**>3.85**	37.41	78.86	1.77	0.79
**>3.86**	36.73	78.86	1.74	0.8
**>3.87**	35.37	79.67	1.74	0.81
**>3.88**	34.69	80.49	1.78	0.81
**>3.9**	34.01	81.3	1.82	0.81
**>3.91**	34.01	82.11	1.9	0.8
**>3.92**	33.33	82.93	1.95	0.8
**>3.93**	32.65	83.74	2.01	0.8
**>3.95**	32.65	84.55	2.11	0.8
**>3.96**	31.29	86.18	2.26	0.8
**>3.98**	29.93	86.99	2.3	0.81
**>3.99**	29.93	87.8	2.45	0.8
**>4.08**	21.09	87.8	1.73	0.9
**>4.09**	20.41	88.62	1.79	0.9
**>4.12**	20.41	90.24	2.09	0.88
**>4.17**	17.01	90.24	1.74	0.92
**>4.19**	17.01	91.87	2.09	0.9
**>4.25**	16.33	94.31	2.87	0.89
**>4.28**	11.56	94.31	2.03	0.94
**>4.29**	11.56	95.12	2.37	0.93
**>4.32**	10.88	95.12	2.23	0.94
**>4.35**	10.2	95.93	2.51	0.94
**>4.37**	9.52	95.93	2.34	0.94
**>4.38**	9.52	97.56	3.9	0.93
**>4.41**	8.84	97.56	3.63	0.93
**>4.47**	8.16	98.37	5.02	0.93
**>4.48**	7.48	99.19	9.2	0.93
**>4.58**	1.36	99.19	1.67	0.99
**>4.64**	1.36	100		0.99
**>5.37**	0	100		1

Supplementary Table 1. Summary table of receiver operating characteristics (ROC) curve results. Sensitivity and specificity are listed along with respective cut-off values and likelihood ratios for ATP5A1 (A), HSP60 (B), PABPC3 (C), ITM2B (D), IF3E1 (E), DBI (F), and MYL6 (G). Results were calculated using the Hanley & McNeil method (1982). +LR (Positive likelihood ratios) are given as a ratio between the probability of a positive result in a positive case and a positive result in a negative case. Similarly, -LR (Negative likelihood ratios) are given as a ratio between the probability of a negative result in a positive case and a negative result in a negative case. Highlighted cells represent optimal criterion value for each protein calculated using the Youden index.

Hanley JA, McNeil BJ (1982) The meaning and use of the area under a receiver operating characteristic (ROC) curve. Radiology 143:29-36.
